# Addiction Recovery: A Systematized Review

**Published:** 2020-04

**Authors:** Mehrnoosh Inanlou, Bahman Bahmani, Ali Farhoudian, Forough Rafiee

**Affiliations:** 1 Department of Counseling, University of Social Welfare and Rehabilitation Sciences. Tehran, Iran.; 2 Substance Abuse and Dependence Research Center, University of Social Welfare and Rehabilitation Sciences,Tehran, Iran; Department of Psychiatry, Roozbeh Hospital, Tehran University of Medical Sciences,Tehran, Iran.; 3 Nursing Care Research Center, Iran University of Medical Sciences, Tehran, Iran.

**Keywords:** *Concept Analysis*, *Definition*, *Recovery*, *Recovered*, *Substance Use Disorders*

## Abstract

**Objective:** Despite the fact that a practical definition of addiction recovery is necessary to conduct an appropriate intervention and research, this concept is still vague and there is no consensus over its meaning and how to measure it. Thus, this study aimed to define and clarify this concept based on the available literature.

**Method** **:** The theoretical part of Schwartz_Barcott and Kim’s Hybrid Model of concept analysis was used to analyze the concept of “Addiction Recovery.” To find the relevant literature, an electronic search on valid databases was conducted using keywords related to the concept of addiction recovery. Medlib, IranMedex, Magiran, SID, Irandoc, Google Scholar, PubMed, Web of Science, Medline, Scopus, Pro Quest, CINAHL, Science Direct, Ovid, and Wiley databases were searched up to December 2018 without a time limitation using the following keywords: “Substance use disorders”, “Drug use”, “Recovery”, “Opioids”, “Addiction treatment”, “Dependency”, “Rehabilitation”, Remission”, “Concept analysis”, “Restore”, “Definition”, “Meaning”, and “Conceptualization”. The Conventional content analysis was used on selected research articles.

**Results: **From a total of 9520 articles, 39 were reviewed and analyzed. Five attributes were selected, including the process of change, being holistic, being client-centric, learning healthy coping, and being multistage. Antecedents are organized into 2 interacting categories: personal and social resources. Personal resources refer to the person, his/her addiction, and the treatment characteristics, while social resources refer to the family, the community, and the context resources. Addiction recovery leads to sustained abstinence, improved physical and psychological health, improved quality of life and satisfaction, meaningful living, and citizenship.

**Conclusion: **These findings may form a basis for the theories, scales, and criteria for the assessment of addiction recovery and will be useful in clinical practices and research. Also, these findings could help health care professionals to understand the concept of addiction recovery, which is important in improving the recovering person in all aspects of rehabilitation. We will report the implementation and analytical phase of this research project, namely, “the addiction recovery concept analysis” in Iran.

Substance use dependence is increasingly recognized as a chronic relapsing condition that may last for decades and requires multiple episodes of care over many years before reaching a sustained state of abstinence (1).The state of substance use and addiction in Iran is unique and linked to specific cultural and social issues (2). There are numerous references to the term recovery in the literature and it is generally defined as an outcome of treating chronic disorders such as addiction (3-5). And what is generally recognized is that recovery refers to more than simply refraining from taking drugs (6). 

In recent years, recovery has been embraced as a target policy in the United States. Similarly, Scotland, England, and Wales consider recovery as a guiding approach to drug policy, and other countries, such as Australia, have considered incorporating this concept into their policies (3) despite the lack of consensus regarding what the term actually means (3, 4, 6-8). 

The advocates of addiction recovery and treatment of the substance use disorders (SUDs) are categorized into 2 groups: the scientific community and the individuals going through rehabilitation. 

Each group advocates a different set of recovery concepts and practices that have been formerly co-functioned. The researchers in the first group, including physicians, SUD (substance use disorder) experts, medical centers, and medical circles, define recovery as a process involving clinical diagnosis, treatment, and rehabilitation (5).

However, researchers who study and assess addiction treatments and addiction policymakers do not have a vivid mental image of recovery (4, 5) despite the recent increase in the popularity of this concept (9). For example, the term recovery has been repeatedly used interchangeably with the words abstinence, remission, and resolution; however, there is no consensus on a unified definition for each one of these terms to differentiate between them (10).

An example of those who have attempted to differentiate recovery from substance use (11) is the Betty Ford Institute Consensus Panel. They differentiated recovery from substance use as a “voluntarily maintained lifestyle characterized by sobriety, personal health, and citizenship.” (4). similarly, in 2008, the UK Drug Policy Commission defined recovery as the “voluntarily sustained control over substance use, which maximizes health, well-being, and contribution in the form of rights, roles, and responsibilities in the society.” A personal transformation process, mirrored in different aspects of performance and fueled by abstinence or increased control over the use of drugs, is at the core of these definitions (8). Deegan (1988) defined recovery as the process of “recovering a new sense of self and of purpose within and beyond the limits of the disability.” (11). All these definitions revolve around abstinence but they are not deemed as the equivalent of recovery.

Experts of addiction treatment usually use the remission (abstinence) criteria set forth in the Diagnostic and Statistical Manual of Mental Disorders (DSM) to differ abstinence from substance use. For instance, the total number of years a person does not suffer from the alcohol use disorder is one of these criteria. According to Dodge et al (2010), this criterion mainly shows the lack of clinical diagnosis of substance use instead of providing a multidimensional frame of reference for recovery (11). 

An addiction recovery model defined within the spiritual framework advocated by Alcoholics Anonymous was proposed by Galanter. The individual’s viewpoint on his/her addiction forms the basis for this model, which reflects the spiritual viewpoint of the Alcoholics Anonymous while covering a major dimension of recovery (12). Chalk, McLellan, and Bartlett have also described recovery with regard to its outcomes, performance, and life quality (11).

The Substance Abuse and Mental Health Services Administration (SAMHSA) assesses recovery through the assessment of an individual’s physical health, mental health, family and social relationships, housing stability, perception of care, access, and retention. This administration introduced a combination of abstinence and improvements in 3 dimensions of the 7 functional dimensions as the sign of recovery (13).

Kaskutas in “What is Recovery?” study argued that the study’s findings illustrate substantial agreement among people who consider themselves in recovery and in how they define recovery. Overall, the six elements endorsed most (>90%) as definitely belonging in their definition included three elements of “essential recovery” (being honest with myself, handling negative feelings without using drugs or alcohol, being able to enjoy life without drinking or using drugs like I used to) and three elements of “enriched recovery” (a process of growth and development, reacting to life’s ups and downs in a more balanced way than I used to, taking responsibility for the things I can change). Although factor scores for these domains were significantly higher among individuals with greater levels of 12-step exposure, the magnitude of differences is small, suggesting that the elements in those factors many of which indeed reflect 12-step principles appear to be somewhat universal among survey participants. Humphreys urged caution not to lose track of the relative agreement among those with lived experience and to avoid definitions so broad that are meaningless or divorced from lived experience (9, 14).

Also, the stigma attached to the substance use disorders can be overcome by communicating the feasibility of recovery. However, this image cannot be cultivated due to the lack of consensus on the definition of recovery (8). Notions such as health, life quality, and chronic disorders have, however, been promoted along with the notion of recovery, and few studies have been performed on the qualities and characteristics of recovery. Therefore, this study was conducted to define and clarify this concept based on the literature to illustrate the concept of addiction recovery through a qualitative analysis and its results have clinical applications.

## Materials and Methods

***Study Design***

In this study, the concept of addiction recovery was analyzed using a hybrid model. The hybrid model concept analysis was used as presented by Schwartz-Barcott and Kim (1993), which amalgamates both theoretical and empirical analysis and is specifically beneficial when exploring a known concept in a new context or when trying to find its new distinctive attribute (2). The model includes 3 phases: First, in the theoretical phase, data are collected using literature reviews to develop a foundation for the second phase. Second, in the field phase, qualitative data are obtained through semi-structured interviews to refine a concept of addiction recovery. Third, in the analytical phase, the concept application and its importance is justified after integration of the data gained during this phase (2). In this study, the theoretical phase which involves searching the literature, dealing with meaning and measurement, and identifying a working definition for the fieldwork phase is presented (2).

***Searching the Literature***

Schwartz_Barcott and Kim (1993) emphasized the extensive need to review the literature. However, it is important to determine the strengths and weaknesses of this definition to generate a tentative description extracted from the literature. The present study employed a systematized and evidence-based approach to search in the literature. This method includes one or more characteristics of a systematic review, but does not claim to present the same results as a systematic review does. The literature was reviewed by focusing on the key question of definition and measurement. The question posed by Schwartz-Barcott and Kims (2000) guided the inquiry through the literature to provide an initial direction for this research (2).

Before beginning the review, a protocol was developed with the following components:

1. The review question: How is addiction recovery described? How can addiction recovery be measured?
2. Article types: The present study reviewed every original article published on the subject of addiction recovery, including quantitative, qualitative, meta-analysis, meta-synthesis, mixed method, and instrument development studies.
3. Search strategy: The search was done using the following keywords: “Substance use disorders”, “Drug use”, “Recovery”, “Addiction treatment”, ”Opioids”, “Dependency”, “Rehabilitation”, “Concept analysis”, “Restore”, “Remission”, “Definition”, ”Meaning”, and “Conceptualization” in electronic databases of Medlib, Iran Medex, Magiran, Sid, Irandoc, Web of Science, Google Scholar, CINAHL, PubMed, Medline, ProQuest, Ovid, and Wiley for articles published up to December 2018, without a time limitation, using the search options provided in each database and EndNote software (X6).

Based on inclusion criteria, articles with full-texts in Persian or English were evaluated by referring to the definition, outcomes, features, and outcomes of the concept of recovery. Exclusion criteria included repeated texts, book reviews, and letters to the editor in languages other than English and Persian.

**Figure 1:**
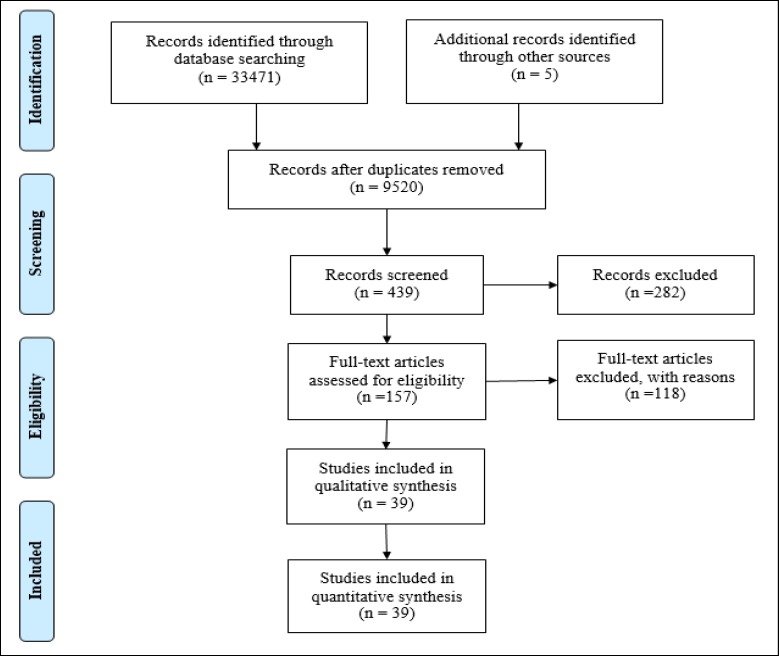
Summary of the Theoretical Phase Based on the PRISMA Flowchart (Selection, Critical Appraisal, Data Extraction of Studies)

After conducting a systematic search using the aforementioned databases, the records retrieved from different databases were saved in Endnote files, which were eventually merged into a shared Endnote file (Figure 1). The relevant articles to the research objectives were found and filtered by analyzing 9520 titles stored in this software. Afterwards, the abstract sections of the selected articles were retrieved. After reading the abstracts of the articles and assessing them based on the research criteria, a total of 437 articles were selected. Then, several electronic magazines were searched to obtain the full-texts of the articles with the reviewed titles. The full-texts of 157 articles were retrieved and analyzed. Eventually, 34 articles matching the research objectives were selected and 5 articles mentioned in the reference sections of some of the articles were included in the study. Therefore, a total of 39 articles were selected. Table 1 presents an overview of studies conducted on addiction recovery. 

***Dealing with Meaning and Measurement***

To analyze the literature, the conventional content analysis method was used based on the model proposed by Graneheim and Lundman. Content analysis refers to the understanding, interpretation, and conceptualization of the core meanings of data. According to Polit and Beck (15), content analysis is the process of organizing and integrating stories and qualitative data that results in the genesis of themes and notions. The text was carefully studied by the researcher as a unit of analysis several times and summarized by meaningful units. Each sentence, phrase, and word referring to the definition and dimensions of the addiction recovery were identified, and each was assigned a code. The codes were categorized by performing continuous comparisons in different categories and subcategories, according to their repetition, differences, and similarities. 

## Results

In this section, the findings from the literature review are discussed in 4 parts: definition of concept, attributes, antecedents, consequences, and method of measurement of the concept.

**1.   Characteristics and definition of the concept**

In general parlance, the word of recovery is defined by Merriam –Webster dictionary as “the process of combating a disorder (such as alcoholism) or a real or perceived problem” (16). Over the last 200 years, various terms have been associated with the resolution of severe alcohol and other drug problems based on conceptualizations of their etiology. These terms have included moral “reformation”, “religious redemption”, “criminal rehabilitation”, or “medical recovery”. In medicine, traditionally, recovery has connoted a return to health after trauma or illness (5, 17). The term recovery has turned into an item of jargon in the state organizations, but it was formerly only linked to 12-step fellowships such as the Alcoholics Anonymous International Fellowship (18).

According to Laudat’s article reviews on recovery, this concept is often defined by the majority of researchers as recovery from drug addiction. It is also commonly defined as a state of abstinence or remission. Furthermore, they define recovery as the process of “overcoming both physical and psychological dependence on psychoactive drugs while making a commitment to society”. Under this definition, recovery includes the acts of avoiding drugs, achieving well-being, and refitting into society (10).

An addiction recovery model defined within the spiritual framework advocated by Alcoholics Anonymous was proposed by Galanter. The individual’s viewpoint on his/her addiction forms the basis for this model, which reflects the spiritual viewpoint of the Alcoholics Anonymous while covering a major aspect of recovery (19).

The American Society of Addiction Medicine (ASAM) made a distinction between recovery and remission. This is done by defining recovery as a state of physical and psychological health, such as one’s abstinence from a dependency-causing drug; and the remission is defined as “freedom from the active signs and symptoms of alcoholism, including the use of substitute drugs during a period of independent living” (5, 19). 

The experimental aspect of recovery has also been addressed in some of the more recent definitions. For instance, “recovery is the experience (a process and a sustained status) through which individuals, families, and communities were impacted by severe alcohol and other drug (AOD) problems and then utilize internal and external resources to voluntarily resolve those problems, heal the wounds inﬂicted by AOD-related problems, actively manage their continued vulnerability to such problems, and develop a healthy, productive, and meaningful life” (20). Although recovery research varies based on how the term has been defined and measured, there is an argument over the application of the term; recovery, abstinence, and remission are used interchangeably. To explore and gain a boarder understanding of recovery, the attribute, antecedents, and consequences of recovery were identified and are described as below. It seems that there is an agreement on the notion that not using substances is at the core of the definition, even if some people may be using a small amount of one substance or another.

**2.   Attributes**

The attributes of a concept are the aspects of that concept used repeatedly to describe that concept and the existence of that concept is contingent. These attributes project a vivid image of the given concept. According to our in-depth analysis of the relevant articles, recovery from drug use is characterized based on the following attributes: 

1-   Process of Change:

Recovery is a process of change, not a static event. Recovery is a continuous and turbulent attempt to maintain abstinence. Recovery refers to an internal and external change in relations, attitudes, thoughts and emotions, or identity change (5, 8, 10, 19, 21-28). 

2-   Holistic:

Given the multidimensional side effects of addiction, recovery is also multiaxial(biological, psychological, social, and spiritual, beyond abstinence) (5, 10, 18, 20, 22, 23). 

3-   Client-Center:

Recovery starts and continues in relation to personal traits, intensity, duration, personal needs, and society. Individuals define their own life goals. In the course of recovery, the individual’s objectives matter and this process may continue at any speed or rate and taking any approach and everyone’s own experience (4, 26, 29-31). 

4-   Learning Healthy Coping Strategy:

Overcoming dependence on substance use and coping with it is an important aspect of recovery. Finding new and better ways to cope with the stressors of life by reaching out for help are important in recovery experience (6, 9, 32, 33).

5-   Multistage: 

Recovery consists of different stages and each stage has its own objectives and interventions (4, 10, 26, 34, 35).

**3.   Antecedents**

**Figure 2:**
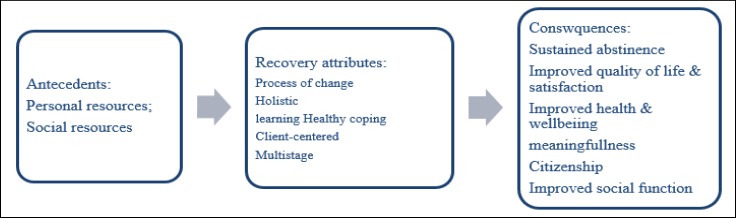
Antecedents and Consequences of Addiction Recovery

In this study, antecedents are those events that should have occurred before recovery (36), which is divided into 2 categories. The “personal resources” and “social resources” are the antecedents of the notion of addiction recovery, affecting this concept in different stages of recovery (Figure 2) (37). The extent and quality of the internal and external resources determine the onset, continuation, and maintenance of complete recovery from addiction. 

1-   Personal Resources:

 A person’s psychological resource, the addiction characteristics, and treatments affect their recovery process. These include self-concept, self-acceptance, belief and skills, attitude, responsibility, hope, openness, honesty, seeing others’ achievements, personal experience, childhood traumas, type and severity of addiction, recovery pathway, coping pattern, personal background, knowledge, and mental and physical health (5, 21, 25, 26, 28, 30, 35, 38-41).

2-   Social Resources:

Recovery is affected by socioeconomic and social factors which involve family and community strengths and responsibilities. Community is the relationships and social networks that provide support, acceptance, friendship, love, respect, and hope. Purpose refers to daily meaningful activities, such as a job, home, school, volunteerism, family caretaking, or creative endeavors, and the independence, income, and resources to participate in the society. Finally, health is overcoming or managing one’s condition and symptoms (20, 21, 23-25, 29, 32, 41-43).

**4.   Consequences**

In this study, consequences were those events that occur as a result of the occurrence (36) of recovery. The consequences of recovery from drug addiction are as follow:

- Sustained control over substance use: Sustained abstinence is an important consequence of recovery.
- Improved physical health: Recovery also improves physical health by mitigating the damage caused by drugs, reducing the feeling of illness, increasing appetite and weight, reducing fatigue and numbness, increasing sexual desire, and restoring the natural temperament and practicing greater self-care (6).
- Improved psychological health: Psychological recovery increases self-worth and self-efficacy; self-acceptance restores identity; and self-perception decreases feeling of shame and guilt, facilitates emotional regulation and management without the need for drug use, helps to gain emotional stability, and brings cognitive reconstruction, and an effective coping mechanism (4, 6, 20, 21, 35, 44).
- Effective citizenship: One of the important consequences of recovery is the respect for our surroundings and others, avoidance of judicial issues, a decrease in crime and leading meaningful lives in the community (4, 6, 9).
- Having a purpose in life: Finding meaning and hopefulness, reconstructing meaning and spiritual growth, and acquiring new values and beliefs such as love, trust, honesty, and acknowledgement can be some of the consequences of recovery from addiction (20, 33, 44, 45).
- Improved social function: Social recovery helps to rebuild and improve social and family relationships, experience more involvement and usefulness in society, improve performance, improve employment and revenue, and experience reunion with the society (4, 6, 9).
- Improved quality of life and satisfaction: Well-being also stems from a decrease in the addiction stigma, an increase in housing stability, an increase in life satisfaction and life quality, and a normal life following recovery (4, 8, 28).

***Measurement***

Valid definition and methods for measuring the recovery are necessary and crucial (46). One of the challenges associated with measuring recovery is that there is no agreement on whether the recovery experience can be standardized or whether it is entirely subjective (47). Close attention to the methods used to measure recovery from substance use reveals that most of the time the question that is used is simply about abstinence in a yes/no format because it is practical (46) or about measuring recovery capital(3) such as “SURE , “ARC” or “ADOM” (30, 37, 47, 48). Measuring recovery from a substance use disorder is much more difficult because recovery is a process, and it is multidimensional(46) and needs to have a multidimensional measure of change and not a single score (10, 46).

**Table 1:** An Overview of Some of Studies Conducted on the Concept of Addiction Recovery

	**Author**	**Country**	**Study Design**	**Study Subject**	**Sample /Sample ** **Size**	**Data ** **Collection**
1	Abedi (2017)	IRAN	Qualitative	Abstinence experience	10 member of NA	Interview
2	BFI consensus panel (2007)	USA	Consensus panel report	Recovery definition		
3	Best et al. (2011)	USA	Mixed method	Recovery Experience	205recovered persons	Questioner- Interview
4	Best etal (2016)	UK	Theoretical paper	Model of recovery		
5	Cano et al.( 2017)	USA	Cross sectional	Recovery capital	546 participants	Questioner
6	Duffy & Baldwin (2013)	UK	Qualitative	Recovery factors	45post recovery persond	Interview
7	Dodge et al. (2010)	USA	Qualitative	Measuring recovery	11professional clinician	Content analysis
8	Dupont et al ( 2016)	USA	Commentary article	Recovery element		
9	Dennis etal (2005)	USA	Cohort	Recovery duration	1271 person in recovery	Questioner
10	Davidosn & white (2007)	USA	Review	Recovery concept		
11	El-gubaly (2012)	Canada	Review article	Recovery concept		
12	Elswik et al (2018)	USA	Qualitative	Recovery exprience	8 in recovery person	Interview
13	Galanter et al (2007)	USA	Instrument psychometric	Spiritual Instrument		Questioner
14	Galanter (2007)	USA	Review	Spirituality in recovery		
15	Laudet (2007)	USA	Mixed method	Recovery mean	440 in recovery persons	Questioner- Interview
16	Laudet& Humphry (2013)	USA	Review	Recovery Concept		
17	Laudet (2008)	USA	Review	Recovery Road		
18	Laudet etal (2006)	USA	Cross-sectional	Recovery factor	353 Recovery person	Questioner
19	Laudet (2002)	USA	Pilot study	Recovery pathway	90 persons	Questioner
20	Law & Guo (2012 )	Taiwan	exprimental	Recovery & hope	40 female drug offender	Questioner
21	Long & Vaughn (1999)	USA	Qualitative	Recovery exprience	7 young person	Interview
22	Grant (2007)	USA	Qualitative	Recovery experience	25in-recovery women	Interview
23	Groshkova (2013)	USA	Focus group	Recovery capital	142 individuals	Questioner
24	Horoosh& Freedman ( 2017)	Israel	Cross sectional	Addiction related growth	104 recovered person	Questioner
25	O Sullivan (2017)	USA	Cross sectional	Recovery capital	76 in recovery persons	Questioner
26	Neal etal (2014)	UK	Focus group	Recovery elements	25professional clinician	Group discussion
27	Neal et al (2015)	UK	Focus group	Recovery measure	46 in recovery person	Questioner
28	Neal etal (2016 )	UK	Focus group	Recovery indicators	124 different member	Group discussion
29	Kaskutas etal (2014)	USA	Internet based survey	Element of recovery	9,341 person in recovery or recovered	Questioner
30	Kelly et al (2015)	USA	Review	Recovery definition		
31	Kearney (1998)	USA	Grounded formal theory	Recovery experience	10 article	Content analysis
32	Kaskutas et al (2015)	USA	Comparative study	Recovery definition	1237 inrecovery person	Questioner
33	Shineborne (2011)	UK	Qualitative	Subjective Experience	6 female	Interview
34	Sterling etal ( 2013)	USA	Cross sectional	Recovery	149 in recovery person	Questioner
35	White( 2005)	USA	Review	Recovery concept		
36	White & Kurtz (2006)	USA	Assay	Recovery Experience		
37	White (2009)	USA	Review	Community Recourses		
38	Witbrodt et al (2015)	USA	Comparative study	Recovery typology	4912 in recovery	Questioner
39	White et al (2006)	USA	Commentary	Recovery factors		

## Discussion

In this study, the concept of addiction recovery was investigated using the theoretical phase of the hybrid model. The results showed that addiction recovery is complex and multifaceted, and a unique process of voluntarily sustained control over substance use which maximizes health and well-being and participation in responsibilities of self, family and community. 

In this study, 5 attributes were extracted as follow: 

One of the attributes was “a process of change”. Recovery is generally considered a journey rather than an incident. Recovery is a process of change, not a static event. Recovery is a continuous and turbulent attempt to maintain abstinence. Recovery refers to an internal and an external change in relations, attitudes, thoughts and emotions, or identity change. Recovery requires the restoration of a currently spoiled identity (5, 8, 10, 19, 21-28). 

The second attribute was being holistic. Given the multidimensional side effects of addiction, recovery is also multiaxial, that is, biological, psychological, social, spiritual, and beyond abstinence. Recovery is multidimensional and is not simply sobriety (11, 49).

The third feature of the concept of addiction recovery is being client-centered: Recovery is self-directed and self-determinant and individualized in nature. Recovery starts and continues in relation to personal traits, intensity, duration, personal needs, and society. Individuals define their own life goals. In the course of recovery, the individual’s objectives matter and this process may continue at any speed or rate and taking any approach, and everyone’s experience is different (22). 

Learned healthy coping was the fourth attribute of the theoretical phase of concept analysis. Overcoming dependence on substance use and coping with the issues it poses are an important aspect of recovery. Finding new and better ways to cope with the stressors of life by reaching out for help are important in the recovery experience. Recovery refers to the way in which a person with addiction or impacted by addiction experience actively manages the disorder or its residual effects in the process of reclaiming full treatment. People feel stronger than prior to the onset of their illnesses (22). 

The fifth attribute was multistage. Recovery consists of different stages and each has its own objectives and interventions. The 3 stages of recovery are as follow: the early recovery stage (3 to 12 months): concentration on the maintenance of abstinence and prevention of relapse; sustained recovery (abstinence lasts from 1 year to 5 years): increased concentration on increasing life stability and attaining goals; and stable recovery (abstinence lasts for more than 5 years): improving the skills and concentrating on growth and development (4). Recovery is a dynamic process characterized by increasing a resulting stable remission and supported by an increased recovery capital and an enhanced quality of life. Recovery is an outcome-based concept. Although previous definitions have suggested some components of recovery, the concept of addiction recovery is wider than recovery solely with abstinence, while including global health, citizenship, quality of life, meaning and satisfaction in life (4, 9, 46, 50). 

As a result, a working definition of addiction recovery was formulated by comparing and contrasting the existing definition with the researchers tentative definition: “Recovery is an intentional endeavor, reclaiming a self-journey, through which a person in recovery with the use of recovery capitals manages the residual drug use effects for sustained control over the substance use, maximizing their health and well-being, having a meaningful life and citizenship, and pursuing other life goals.”

This means that recovery leads to changes, preserves the desired new lifestyle, and incorporates these factors into daily life.

## Limitation

One of the limitations of this study was the lack of access to the full-text copies of some papers. In addition, access to all resources via an electronic database was not possible (eg, Psych INFO). Another limitation was the language barrier and the use of only the Persian and English articles in the literature.

## Conclusion

This finding can provide an insight for researchers to clarify the definition of recovery before designing the research. Ambiguity in the definition of recovery occurred when the researcher tried to emphasize the theoretical differences in its definition but overlooked them in practice. We need to relinquish certain restrictions of the theoretical definition, but instead exert them practically. The researchers and clinicians need to agree on the criteria that determine the indicators of recovery in persons for assessing the recovery. The clarity of language is also a major determinant of our success in making interventions in families, communities, individuals, and AOD cases. Commonly, the findings may help health care professionals to understand the concept of addiction recovery, which is important in making improvements in all aspects of recovery.not only abstinence. 

Finally, our findings may form the basis for the theories, the scales, and the criteria for the assessment of addiction recovery. The results of our concept analysis revealed that many personal and social factors were affected with regard to the person in recovery and the recovery process, as this phenomenon is multidimensional. Health care professionals and clinicians should be aware of the different suitable approaches that should be taken to promote and maintain recovery. The imbalance of power between a person in recovery and clinicians and the focus on abstinence is another important issue and can be resolved by focusing on the definition. We will report the analytic phase of this research project as an addiction recovery concept analysis in Iran.
